# Advancements in MAFLD Modeling with Human Cell and Organoid Models

**DOI:** 10.3390/ijms231911850

**Published:** 2022-10-06

**Authors:** Shi-Xiang Wang, Ji-Song Yan, Yun-Shen Chan

**Affiliations:** 1 Guangzhou Laboratory, No. 9 Xing Dao Huan Bei Road, Guangzhou International Bio Island, Guangzhou 510005, China; 2 School of Life Sciences, Yunnan University, Kunming 650500, China

**Keywords:** MAFLD, human model, cell culture, organoid, microfluidics

## Abstract

Metabolic (dysfunction) associated fatty liver disease (MAFLD) is one of the most prevalent liver diseases and has no approved therapeutics. The high failure rates witnessed in late-phase MAFLD drug trials reflect the complexity of the disease, and how the disease develops and progresses remains to be fully understood. In vitro, human disease models play a pivotal role in mechanistic studies to unravel novel disease drivers and in drug testing studies to evaluate human-specific responses. This review focuses on MAFLD disease modeling using human cell and organoid models. The spectrum of patient-derived primary cells and immortalized cell lines employed to model various liver parenchymal and non-parenchymal cell types essential for MAFLD development and progression is discussed. Diverse forms of cell culture platforms utilized to recapitulate tissue-level pathophysiology in different stages of the disease are also reviewed.

## 1. Introduction

### 1.1. MAFLD Epidemiology

MAFLD is a chronic liver disease composed of a spectrum of liver pathology primarily driven by the accumulation of fats in the tissue [1]. MAFLD patients are categorized by disease severity, including steatosis with no signs of liver injury, non-alcoholic steatohepatitis (NASH) with detectable levels of liver injury and various degrees of fibrosis, and end-stage cirrhosis [2]. MAFLD patients are often associated with a high risk for various metabolic diseases, such as type II diabetes mellitus (T2DM), cardiovascular disease (CVD), and chronic kidney disease (CKD) [3,4]. The condition is also widely considered a hepatic manifestation of metabolic syndromes [5]. MAFLD is proposed to be a more accurate nomenclature than ‘NAFLD’ given the disease’s inextricable link with metabolic syndromes [6]. This review uses MAFLD when referring to all previous NAFLD studies. Globally, 1 out of 4 adults and 3–10% of children and adolescents develop MAFLD, and numbers will likely increase to 31% in adults by 2030 [7,8,9]. Moreover, in 2015, an estimated 20% of all MAFLD patients developed NASH, and the cases will likely increase to 27% by 2030 [9]. Patients with MAFLD and more severe liver fibrosis (stages F3 and F4 or cirrhosis) have an estimated higher risk of liver-related complications and deaths compared with the control group (fibrosis stages F0 to F2) [5,10]. The high incidence of MAFLD supports a pressing need for treating this metabolic disorder. However, no approved drug treatment is available for MAFLD patients [11,12]. Given the close pathophysiological link between MAFLD and type 2 diabetes (T2DM), drug development companies are actively exploring T2DM therapeutics such as thiazolidinediones, GLP1R glucagon-like peptide 1 receptor (GLP1R) agonists, farnesoid X receptor (FXR) agonists, and sodium–glucose cotransporter-2 (SGLT2) inhibitors for MAFLD treatment in ongoing human clinical trials [11]. Among the challenges faced in MAFLD drug discovery, developing physiologically relevant human models for mechanistic studies and screening of therapeutics remains a significant hurdle. 

### 1.2. Genetic and Environmental Factors Driving MAFLD

The growing numbers of MAFLD-related studies have identified genetic and environmental factors responsible for disease development and progression. The increased risk factors observed in first-degree relatives and monozygotic twins support the heritability of MAFLD [13,14]. Genome-wide association studies (GWAS) in multiple NAFLD cohorts have identified risk variants in genes, including patatin-like phospholipase domain-containing 3 (*PNPLA3*), membrane-bound O-acyltransferase domain-containing 7 (*MBOAT7*), transmembrane 6 superfamily member 2 (*TM6SF2*), apolipoprotein C3 (*APOC3*), and glucokinase regulator *GCKR* [15,16]. Conversely, a protective single-nucleotide polymorphism (SNP) variant on the *HSD17B13* gene locus was identified in an independent cohort [17]. This gene encodes for a poorly characterized enzyme (17-beta hydroxysteroid dehydrogenase 13) in the hepatocytes, and the reported mutation generates a truncated enzyme. Mechanistic insights on how this variant confers protective functions for MAFLD will likely reveal novel therapeutic targets. While population genetic studies across the globe identify more disease-associated mutations, much remains to be uncovered on how these reported genetic risk variants function. *PNPLA3* is one of the earliest and most widely reported SNP variants identified in multiple independent cohorts, and how the encoded enzyme contributes to elevated MAFLD risk remains unclear. Conflicting reports of *PNPLA3* contribution to hepatic steatosis in human in vitro models [18,19] and mouse in vivo models [20,21] highlighted the potential need to refine current human MAFLD models. 

Similar to other metabolic syndromes and diseases, non-genetic drivers of MAFLD development and progression include sedentary lifestyles and nutrient-excessive diets. A high intake of fats and carbohydrates and a lack of physical activity promotes the excessive accumulation of lipids in the liver [22,23,24]. Notably, fructose intake correlated with fibrosis severity in MAFLD patients [23]. In comparison to glucose, fructose uptake and metabolism in the hepatocytes are relatively unregulated and, in the process, generate up to 100-fold more reactive oxygen species (ROS) production in the cells [25]. Besides being a substrate and inducer of de novo lipogenesis (DNL), fructose increases ER stress in the hepatocytes and stimulates pro-inflammatory response. A ‘two-hit’ or ‘multiple-hit’ hypothesis has been proposed for steatosis progression to NASH, where the buildup of fats in the hepatocytes prime the cells to inflammatory events stimulated by other agents such as bacterial toxins or metabolites [26,27]. Besides the high levels of nutrients supplemented in the MAFLD-associated ‘Western diet,’ the variety of food intake influences the gut microbiome, which strongly correlates with MAFLD progression and severity [28,29,30]. Metagenomic profiling of MAFLD patients at different stages by Loomba and colleagues identified microbial features uniquely enriched in MAFLD patients with advanced fibrosis [28]. In an independent study, Caussy et al. reported a similar phenomenon in MAFLD patients with cirrhosis [30]. To further understand how the microbiome plays a role in MAFLD development, Hoyles et al. performed a multi-omics study including metagenomics and phenomics profiling in obese patients with and without MAFLD [29]. The authors reported that the guts of obese individuals with early-stage MAFLD are low in microbe diversity and enriched for specific bacteria phyla such as Actinobacteria and Proteobacteria. Pathway analysis further identified corresponding increases in branched-chain amino acid (BCAA) and aromatic amino acid (AAA) metabolism [29]. Notably, the author demonstrated that phenylacetic acid (PAA), a microbial metabolic product of phenylalanine (an AAA), is enriched in the serum of the obese patient with steatosis, and PAA treatment induces MAFLD development in human hepatocytes and mice [29].

### 1.3. Molecular and Cellular Features of MAFLD Progression

**Figure 1:**
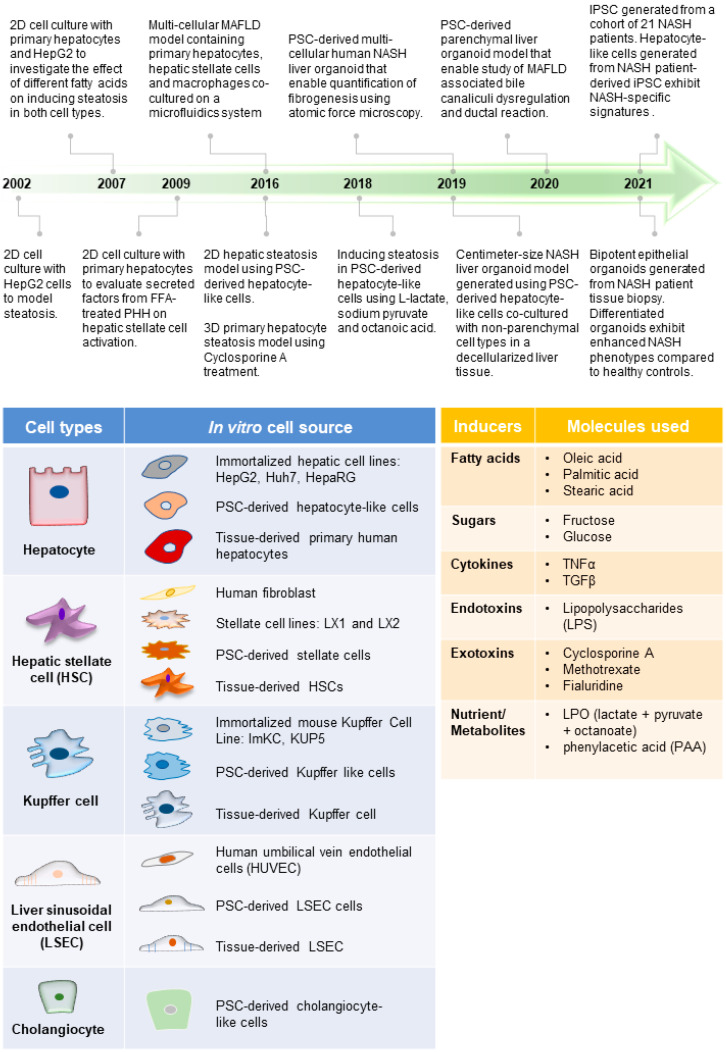
**Advances in MAFLD human models**. (Top) Timeline of representative MAFLD human model studies [41,42,43,44,45,46,47,48,49,50,51,52], which reflect how different cell types and the inducers of MAFLD (tables below) have been employed to create increasingly complex model systems. This includes the use of various cell culture platforms such as organoid culture systems as well as microfluidics. (Bottom) Tables listing the commonly used cell types for modeling different liver cells and molecules used in various studies to induce MAFLD phenotype (inducers).

**Figure 2:**
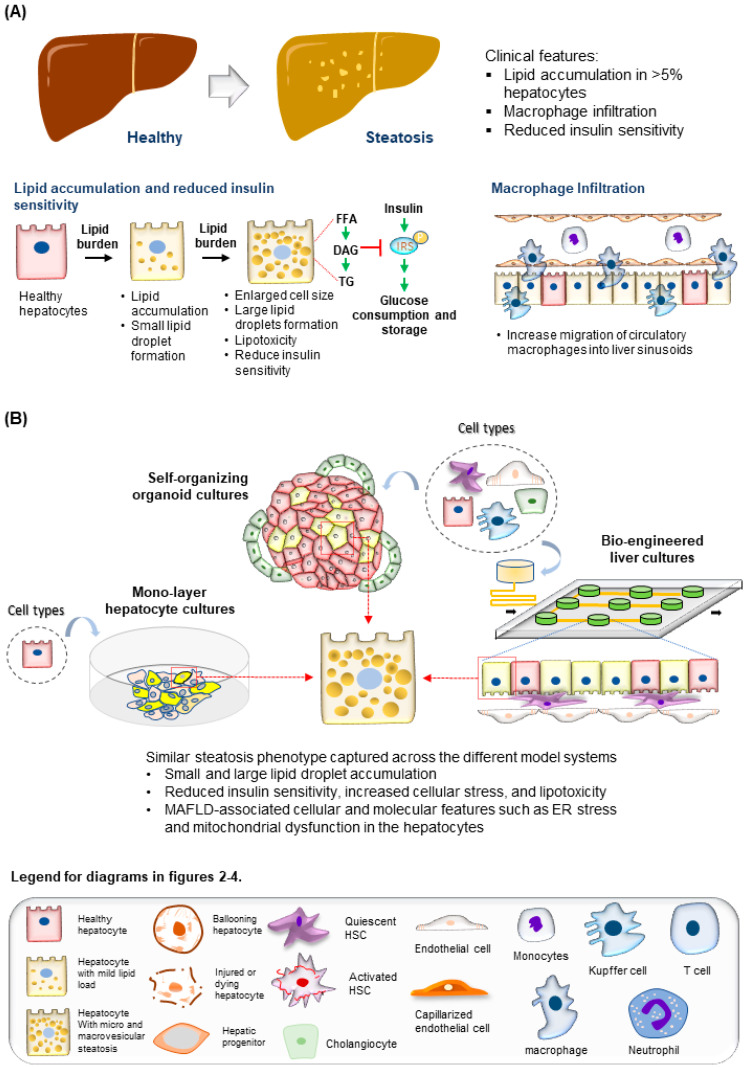
**Modeling steatosis development**. (**A**) (Top) Tissue features observed in liver biopsies from patients with steatosis. (Bottom) The major molecular and cellular changes during steatosis development in the liver. (**B**) (Top) Steatosis phenotypes observed in the hepatocytes are well recapitulated using various culture systems discussed. (Bottom) Legend for diagrams in Figure 2, Figure 3 and Figure 4.

The liver is composed of multiple cell types, including the parenchymal hepatocytes and cholangiocytes, as well as the non-parenchymal cells (NPCs) such as the liver sinusoidal endothelial cells (LSECs), hepatic stellate cells (HSCs), fibroblasts, and Kupffer cells (Figure 1) [31]. The cells are spatially arranged in the liver sinusoids to form networks of fluid channels such as the sinusoidal capillary and bile canaliculi. These various cell types and tissue structures are affected as MAFLD develops and are involved in disease progression at different stages [2]. The initial phase of MAFLD, hepatic steatosis, is defined by fat infiltration in more than 5% of the hepatocytes in the liver with no significant liver injury and fibrosis detected [5]. The flux of excessive non-esterified fatty acids (NEFAs) into the liver results in the visible accumulation of lipid vesicles of different sizes in the hepatocytes (Figure 2) [32,33]. The increased lipid species in the steatotic hepatocyte also reduces insulin sensitivity, a prominent molecular dysregulation in MAFLD progression [34,35]. Evidence from pre-clinical and clinical studies has shown that increased diacylglycerol (DAG) in hepatocytes upregulates protein kinase-Cε (PKCε) activity [36,37], which in turn inhibits the insulin signaling pathway by reducing phosphorylation of insulin receptor substrate-2 (IRS2) and phosphatidylinositol-3-OH kinase PI(3)K [38]. Insulin resistance in the cell, in turn, drives steatosis development by promoting DNL through increased glucokinase activity and impaired glycogen synthase activation. During disease progression, oxidation of the excess fatty acids increases ROS generation [39], and reduced glucose supply can further aggravate the process [40]. The increased ROS species promotes lipid peroxidation, resulting in the injury of organelles and the cellular membrane, and promotes apoptosis. 

**Figure 3:**
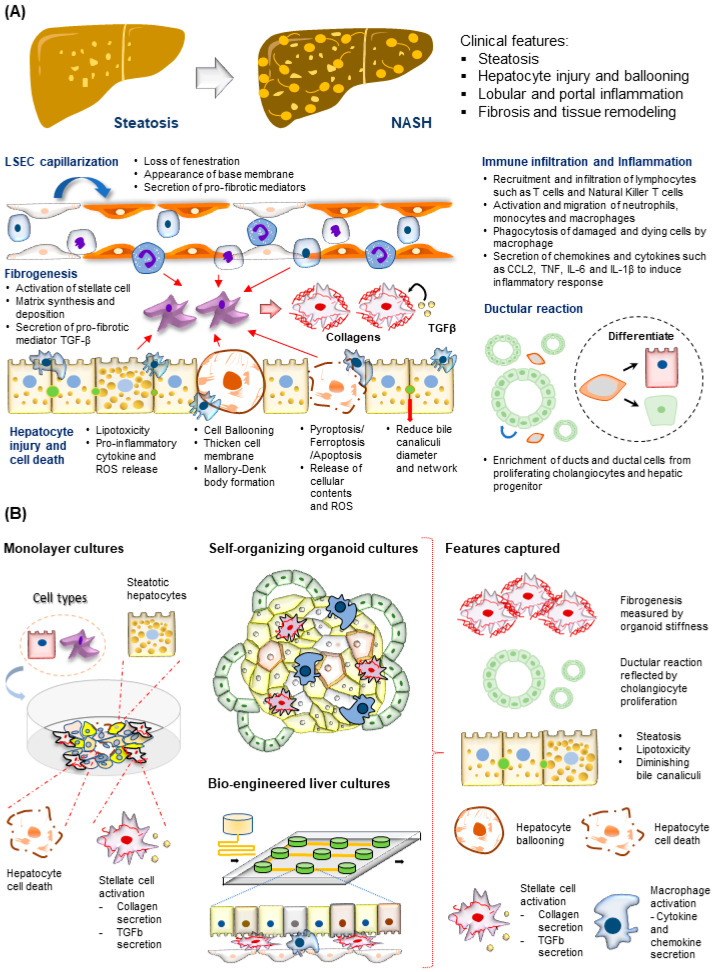
**Modeling NASH development**. (**A**) (Top) Tissue features observed in liver biopsies from patients with NASH. (Bottom) The major molecular and cellular changes during NASH development in the liver. This includes hepatocyte injury and cell death, immune cell infiltration and inflammation, fibrogenesis, LSEC capillarization, and ductular reaction. (**B**) (Left) NASH-associated cellular changes captured using monolayer cultures of hepatocytes and HSC. (Right) NASH-associated cellular and structural changes observed in the hepatocytes recapitulated using various 3D culture systems described.

The time taken for progression from steatosis to NASH varies among individuals and could take up to 14 years [53]. This lengthy period supports a dynamic interplay of interdependent inflammation and fibrogenesis activities, resulting in increased hepatocyte injury, cell death, and liver niche remodeling (Figure 3A). Other cell types involved in this process include both the innate and adaptive immune cells, such as macrophages, neutrophils, and T cells [54,55], and stromal cells, such as LSECs [56] and HSCs [57]. Significant drivers of inflammatory events include microbial pathogen-associated molecular patterns (PAMPs) from the gut, death-associated molecular patterns (DAMPs) released from apoptotic hepatocytes, immune-cell-released chemokines such as CXCL2 and CXCL5, and cytokines including TNFα, IL6, and IL10 [58,59,60]. The portal infiltration of immune cells such as macrophages can already be detected in the biopsy of the steatotic liver, supporting the involvement of immune cells at the early stages of disease progression [54]. A converging downstream effect of these inflammatory responses is the activation of the quiescent HSC, which plays a central role in remodeling the extra-cellular matrix (ECM) to promote fibrogenesis (Figure 3A) [61]. Major events include the PDGF-mediated HSC proliferation and migration to injury sites and TGFβ stimulation of HSC secretion of fibril-associated collagens and matrix metalloproteinase (MMP). These activities gradually remodel the soft and perforated endothelial/epithelial basal lamina to a stiff and obscure one rich in fibril-forming collagen types I and III. As fibrogenesis progress in NASH, the collagen fibrils thicken and form vast networks and septa that increasingly disrupt normal liver functions. Fibrogenesis remains the only histological feature predictive of clinical outcomes in NASH patients [62]. Besides inflammation and fibrogenesis, hepatocyte injury assessed by ‘hepatocellular ballooning’ is a defining pathological feature indicating disease progression from NAFLD to NASH (Figure 3A) [63]. These ‘ballooned’ hepatocytes have an enlarged cell size with a thickened cell membrane, rarefied cytoplasm, and the presence of the Mallory–Denk body [64,65]. Recapitulating these molecular and cellular features is essential for creating physiologically relevant human MAFLD models.

### 1.4. In Vitro Human Models for MAFLD

Animal models of MAFLD have been widely reported and used in drug evaluation studies [22,66]; however, significant species-specific differences between human and animal livers potentially hindered the clinical translation of discoveries generated from animal models [67,68]. The development of human in vitro MAFLD models is critical to providing a parallel platform to investigate human cells’ specific mechanisms and treatment responses. Over the past two decades, significant progress in creating human models of MAFLD in a dish has been achieved (Figure 1).

Researchers have engaged the use of increasingly complex culture systems to recapitulate different pathophysiological features in various stages of MAFLD. The models can be broadly classified into simple monolayer cultures composed mainly of single cell types (Table 1) or complex 3-dimensional (3D) cultures consisting of single or multiple liver cell types (Table 2). Various cell sources mimicking endogenous liver parenchymal cells and NPCs and inducers of lipid accumulation, including nutrients and metabolites, have been employed [29,41,42,69,70,71] (Figure 1). Monolayer cultures of human primary hepatocytes or immortalized cell lines provide a simple approach to model early hepatic cellular response under excessive free fatty acid (FFA) exposure (Table 1 and Figure 2B). However, MAFLD progression involves multiple cell type interactions and remodeling of the tissue environment [61]. Modeling such events during MAFLD progression requires more sophisticated cultures containing various liver cell types and the recapitulation of the cellular spatial organization (Figure 3A,B). Recent progress in bioengineering approaches and 3D cell culture techniques have further enabled the creation of such sophisticated models (Table 2). In this review, we aim to provide a comprehensive summary of current efforts to model MAFLD, focusing on human in vitro models. We also discuss the limitations of existing platforms and future advancements required to better model MAFLD in a dish.

## 2. Human Immortalized and Primary Cell Lines for Modeling MAFLD

Human hepatic cells widely employed in MAFLD studies include immortalized hepatocytes, cancer cell lines, primary hepatocytes, and pluripotent stem cell (PSC)-derived hepatocyte-like cells (HLCs) (Table 1). The monolayer culture of these cell lines is primarily used to model hepatocyte uptake of lipids, including the transport, storage, and metabolism of the excess lipids. In addition, the homogenous cell culture is suitable for dissecting direct molecular and cellular responses of hepatocytes exposed to nutrition and environmental changes in the liver through manipulating the cell culture media. The stable cell lines also allow further genetic perturbations for gain and loss of function studies.

### 2.1. Immortalized Hepatic Cell Lines for Modeling MAFLD

One of the most widely used hepatic cell lines for modeling human MAFLD is the HepG2 cell line derived from hepatocellular carcinoma (HCC) and subsequently immortalized for stable culture in vitro [85]. HepG2 cells express and secrete a variety of major hepatic plasma proteins, including albumin, and exhibit several hepatocyte-specific responses to environmental stimuli, making the cells suitable for in vitro modeling of hepatocyte functions [86,87]. Several treatment regimens and detection assays have been widely adopted to induce and measure steatosis in HepG2 cells. Treatment of HepG2 cells with oleic acid (OA), a monounsaturated fatty acid, could cause the accumulation of intracellular triglyceride and lipid droplets within the cells. These phenotypic changes can be easily detected by staining with lipid dyes such as Oil red or Nile red and quantified with lipid assay kits [43,44,72]. On the other hand, OA treatment is insufficient to induce changes in the cellular oxidative phosphorylation state, which was dysregulated in NASH patients [88]. This phenotype was recapitulated by co-treatment with saturated fatty acids, such as palmitic acid (PA) or stearic acid (SA) [73,74]. HepG2 cells treated with different ratios of OA to PA develop varying steatosis phenotypes; a higher proportion of OA results in benign chronic steatosis, while a higher PA proportion induces more significant toxic and apoptotic effects in the cells [44]. In addition, PA treatment induces the production of pro-inflammatory chemokine IL-8 in HepG2 cells, recapitulating the elevated IL-8 levels seen in NASH patients [73,89,90]. This simple system is also employed for mechanistic MAFLD studies and the validation of therapeutics [75,91]. C1q/TNF-related protein 9 (CTRP9) is an adiponectin paralog expressed by adipose tissue, and overexpression of this protein has been shown to reduce steatosis in mouse models [92]. To further unravel how CTRP9 functions in the human hepatocyte, Jung et al. utilized PA-treated HepG2 cells which similarly exhibited reduced hepatic steatosis upon CTRP9 treatment. The authors unravel that CTRP9 induces protective effects against steatosis through the inhibition of ER stress via the activation of AMPK-mediated induction of autophagy [91]. 

A similar immortalized cell line of growing interest for MAFLD modeling is HepaRG [78,79]. These cells exhibit greater sensitivity to drug-induced steatosis [78] and features of MAFLD upon treatment with free fatty acids [79]. Other immortalized hepatic cell lines, such as fetal liver-derived WRL-68 and HCC-derived Huh7 cells, are also used in MAFLD modeling (Table 1) [69,76,77]. All these immortalized cell lines have provided a simple approach to modeling the disease, and the ease of culture and high cell viability have made them excellent cell options for engineering approaches to create more complex cellular models. However, these cells may not fully reflect the primary hepatocyte due to the cancer origins of some of the cell lines and the cellular changes induced by the transgenic approaches used to immortalize the cell. The altered hepatic nature of the cells raised concerns about the accuracy of observations from MAFLD models generated with these cells [85,93]. 

### 2.2. Liver-Tissue-Derived Primary Cells for Modeling MAFLD

Primary human hepatocytes (PHHs) derived from liver tissue have been a source of physiologically relevant in vitro models for liver diseases, including MAFLD [41,44,45,70,73,80,81,91,94,95,96]. The PHH culture faithfully retains many molecular and cellular features of their in vivo counterparts and the disease phenotypes of the liver origin. Similar to HepG2 cells, PHHs respond differently to the varying ratios of OA and PA used in treatment [44]. The differential response to OA and PA was validated by analyzing the cell viability, gene expression, and secretome of treated cells [44,45,80,81,91,95,96]. Treatment of PHHs with PA induces triglyceride accumulation, elevated expression of lipogenic genes, a significant increase in IL-8 release, and increased ER stress [73,91,96,97,98]. In addition, BMP-8B was induced in OA-treated PHHs, correlating with observed upregulation in the human steatotic liver tissue [81]. PHH MAFLD models were also employed to evaluate therapeutic modalities such as CTRP9 and Humanin to alleviate the steatosis phenotype induced by FFA treatment [91,96]. However, the lack of self-renewing capacity and a limited number of cells that can be derived from a single liver poses significant challenges to using PHHs in large-scale applications. The limited PHH source also results in a high cost, further restricting their adoption in MAFLD studies. More importantly, the molecular and functional differences observed in hepatocytes from different individuals may influence the reproducibility of results. Prill et al. evaluated the FFA treatment response of hepatocytes derived from five donors where two donors had the reported TM6SF2 E167K genetic variant [82]. While the study validated the effects of the TM6SF2 E167K genetic variant in conferring susceptibility to steatosis, the results also highlight variability in FFA treatment response observed across different donor hepatocytes. Idiosyncratic responses have been a significant limitation for using these cells for liver toxicology and disease modeling [99]. 

To overcome the abovementioned limitations, scientists have attempted to isolate expandable primary hepatic progenitors from human liver biopsies [100,101,102]. Clever’s group first reported the successful expansion of bipotent cholangiocyte progenitors from human liver tissue, which can differentiate into functional hepatocytes in vitro [100]. The group subsequently optimized the protocol to achieve the culture of expandable human hepatocyte progenitors that can more efficiently generate hepatocytes with improved functions and engraftment efficacy [102]. In parallel, several groups have also reported the successful derivation of expandable hepatic progenitors from isolated human hepatocytes [103,104] and human fetal liver, respectively [105]. Similarly, these expandable progenitors are a renewable cell source for generating functional primary hepatocytes. In addition, as the cells can expand and maintain their progenitor state, transgenic cell lines can be generated with genome editing tools to facilitate the study of gene functions [106,107]. McCarron et al. employed the cholangiocyte progenitor culture method [100] and generated NASH patient-specific models from their liver biopsies [46]. The cells derived from NASH patient tissue exhibit significant dysregulation in lipid metabolism and hepatic functions. They also express pro-inflammatory and fibrogenesis-associated genes and have reduced proliferative capacity. Of concern, these observations are idiopathic and not captured across cell models from different NASH patients. Intriguingly, the authors also showed that the NASH patient-derived cells express high levels of Ubiquitin D (UBD), an inhibitor of RNA virus-induced interferon signaling, and hypothesized that they are more susceptible to virus infection. The authors demonstrated that the NASH patient-derived cell models can be more easily infected by SARS-CoV-2 pseudovirus (vesicular stomatitis virus (VSV) expressing SARS-CoV-2 spike protein), correlating with the reports of more severe COVID-19 infections in NASH patients [108,109]. Despite these breakthroughs, significant challenges exist for the wide adoption of this liver progenitor culture system. In the clinic, the invasive nature, high cost of liver biopsies, and limited drug treatment options dissuade MAFLD patients from undergoing the surgical procedure [110]. This limits the accessibility of patient liver tissue for establishing the cell cultures. There are also significant hurdles for each progenitor culture system [100,102]. The cholangiocyte progenitor culture system, which enables efficient derivation and long-term culture of cells from the NASH patient [100], is inefficient in generating functional hepatocytes [102]. On the other hand, the hepatocyte progenitor culture system, which efficiently generates highly functional hepatocytes, could only be stably derived from fetal tissues [102].

### 2.3. PSC-Derived Hepatic Cells for MAFLD Modeling

PSCs such as embryonic stem cells derived from blastocysts or induced pluripotent stem cells (iPSCs) generated from adult somatic cells are highly renewable cell sources with the capacity to differentiate into cell types of all three germ layers [111,112]. Harnessing the PSC’s ability to generate endoderm lineage cell types, including lung, gastrointestinal tract, liver, and pancreas, multiple groups have established protocols to derive functional hepatocytes and cholangiocytes from PSCs in vitro [113,114,115,116,117,118,119,120,121]. While these PSC-derived hepatocytes expressed cell-type-specific markers such as ALB, CK8, HNF4A, and CYP3A4, the PSC-derived cells are fetal in nature. The cells express high levels of alpha-fetoprotein (AFP), not found in healthy adult liver tissue. They also lack mature hepatocyte features and functions, including the absence of mature cytochrome proteins and response to drug treatment [122]. Hence, these cells are often referred to as HLCs. These PSC-derived HLCs can be utilized in various primary hepatocyte-related applications, from modeling liver development and diseases [47,114,123,124,125,126] to cell-based regenerative applications [127]. In modeling MAFLD, HLCs treated with FFAs could accumulate lipid droplets, exhibit cellular stress responses, express genes associated with lipid metabolism, and recapitulate transcriptional profiles similar to MAFLD liver tissues [19,47,48,49,83,84]. The self-renewing capacity of PSCs enables scalable production of HLCs for applications requiring a large number of cells. Harnessing this inherent advantage, Parafati et al. utilized PSC-derived HLCs in a 13,000-compound high-throughput screen to identify molecules that can reverse ER-stress-induced steatosis [83]. The study revealed that cyclin-dependent kinase (CDK) inhibitors can attenuate steatosis through the cyclin D3-CDK2-4/CCAAT-enhancer-binding proteins/diacylglycerol acyltransferase 2 pathway. 

An inherent advantage of modeling disease using the iPSC platform is the generation of genetic models through genome editing of iPSC cell lines or the establishment of iPSC lines from patients with the genotype of interest [49,112,128,129]. To dissect the role of PNPLA3, the most widely reported gene associated with the full spectrum of MAFLD, Tilson et al. generated *PNPLA3* knockout and PNPLA3 I148M variants in isogenic iPSC cell lines using the CRISPR genome editing tool [19]. In contrast to the mouse model with similar genetic edits, the human HLCs with depleted PNPLA3 and I148M variants demonstrated increased steatosis with significant improvements in cell viability under saturated fatty acid treatment. Furthermore, the authors revealed that PNPLA3 depletion and expression of the I148M variant in the cells increased polyunsaturated fats and altered lipid metabolism profiles, which correlated with patient tissue profiles. While these changes protected the cells from lipotoxicity, they also sensitized them to drug- and alcohol-induced injury. This study highlighted the main advantages of MAFLD studies with human cellular models, where genetic manipulations and biochemical assays with homogenous human hepatocyte cultures are advantageous for mechanistic studies. A parallel study by Duwaerts et al. further highlighted the iPSC platform’s potential for unraveling novel genetic drivers of MAFLD by generating patient-specific MAFLD models [49]. The team established 21 iPSC cell lines from MAFLD patients, including 18 NASH patients. Intriguingly, iPSC-HLCs generated from NASH patients had increased lipid droplet accumulation and expressed distinct transcriptomic profiles compared to iPSC-HLCs generated from individuals without MAFLD. Dysregulated genes are associated with cell death and transformation, insulin resistance, and cellular oxygen consumption. This observation supports the existence of genetic or epigenetic drivers of the MAFLD phenotype in the iPSC cell lines. Further in-depth genomics study and expansion of sample sizes would likely facilitate the identification of MAFLD regulatory genes and mutations. Importantly, this study underlines the potential of generating patient-specific MAFLD models for drug testing, establishing the foundation for future precision therapy. 

The advent of cell fate programming technologies has also enabled the derivation of hepatic cells from other cell sources, including skin cells and fibroblasts [117,118,130]. The trans-differentiation or direct cell fate programming approach avoids the reprogramming to the embryonic cell state, which accounts for the fetal phenotype of iPSC-derived HLCs. On the other hand, whether these trans-differentiated hepatocytes could provide additional insights for MAFLD modeling remains to be seen. 

### 2.4. Liver Non-Hepatic Parenchymal Cell Types 

Besides the hepatocytes, NPCs play an integral role in the progression of MAFLD (Figure 3A). The role of resident macrophages (Kupffer cells) and infiltrating circulatory macrophages in early and late MAFLD development has been widely reported [131]. Besides regulating inflammatory responses during NASH progression, chemokines and cytokines released by macrophages have also been reported to promote steatosis in hepatocytes and induce HSC migration and activation. MAFLD studies using immune-deficient mice identify the need for immune cells such as T and NK cells in modulating NASH development [66,132]. Histological analysis of NASH patient tissues also identified changes in the T cell population, including increased T helper 17 (TH17) cells and decreased regulatory T (Treg) cells [133]. NK cells are also reportedly reduced in NASH liver tissue [55]. In recent years, other immune cells, including neutrophils [134] and platelet cells [135], are also identified to play a role in MAFLD development, prompting a need for such human cell types in in vitro models (further discussed below). On the other hand, HSCs and hepatic fibroblasts are essential cell types responsible for fibrogenesis development during NASH progression. During MAFLD development, capillarization of LSECs is also observed [136]. During this process, fenestration in the LSEC is gradually closed, and the basement membrane thickens, reducing the elasticity and permeability of the capillaries. These changes reduced chylomicron uptake by the hepatocytes for VLDL production, thereby increasing cholesterol and triglyceride productions which promote steatosis. The dysregulated LSECs also contribute to the pool of oxidants and inflammatory signals that further promote inflammation and fibrosis development. Primary cells from human and mouse tissues, as well as immortalized and PSC-derived cell lines that resemble these NPCs, have been used in co-cultures to model liver inflammatory and fibrosis events [45,70,137,138,139,140,141,142,143,144,145,146,147,148,149,150], including MAFLD models (Figure 1). 

## 3. Human Multi-Cellular 3D MAFLD Models

MAFLD progression requires the crosstalk of multiple cell types spatially arranged in specific layers and orientations (Figure 3A). Cells are often cultured in 3D to promote the formation of tissue-like structures. The cell sources are similar to those described above in monolayer cultures (Table 1). Strategies reported to generate 3D MAFLD models can be divided into two general categories: self-organizing liver organoid models and bioengineered liver models (Table 2). Liver organoids discussed in this review are defined as 3D cultures of single or multiple liver cell types that exhibit liver functions (Table 2). Some liver organoids described can recapitulate tissue structures [47]. The organoid formation mainly depends on cell–cell interactions among cell types used, and the outcome is less controlled with higher heterogeneity among replicates. In contrast, the bioengineering approach is employed to further control cell interactions and orientations to generate larger and more complex structures with higher reproducibility. On the other hand, bioengineering approaches are difficult to adopt due to the availability of technology and operational expertise required. In this review, we will cover major studies utilizing these approaches to generate tissue-like human MAFLD models. 

### Self-Organizing Liver Organoid Model

One of the most adopted strategies in creating human organoid models is the stepwise differentiation of PSC along the targeted lineage. This approach harnesses the self-organizing capacity of the progenitor and differentiated cells to form interactions essential to creating structures that mimic human tissue. Multiple groups have generated liver organoids from PSCs for various applications [151], including MAFLD disease modeling (Figure 3A,B) [47,50,126]. Ouchi et al. reported one of the first multi-cellular organoid cultures generated from PSCs [50]. Single-cell analysis shows that the PSC-derived organoid contains multiple cell types resembling hepatocytes, cholangiocytes, HSCs, and Kupffer cells. The ability to generate various cell types from a single starting cell population highlights the strength of using PSCs. The main advantage of this reported strategy is the relatively short duration needed to generate the liver organoids (~20 days) and induction of the inflammatory and fibrosis response in the liver organoid using OA treatment (~5 days). Within three days of treatment, the authors could observe inflammatory reactions from detecting secreted IL-6 and upregulation of *TNFα* and *IL-8* expression. A key highlight of this study was modeling FFA-induced fibrogenesis and employing the model for drug response study. The authors can detect upregulation of conventional fibrogenesis markers, including type III procollagen peptide (P3NP), VIMENTIN, and α-smooth muscle actin (α-SMA) in the FFA-treated organoids. In addition, the authors were able to detect increased stiffness in the organoid using atomic force microscopy (AFM)-based live indentation measurement. The authors determined the elasticity of each organoid and the range of Young’s modulus (Pa) measured increase when organoids are treated with increasing levels of lipopolysaccharide (LPS) and OA. This platform opens a novel avenue for modeling fibrogenesis during MAFLD progression and potentially a high-throughput screening platform for drug screening studies, given the scalability of PSC culture. On the other hand, there is a need for more biochemical studies to ensure that metabolic changes within the hepatocytes during MAFLD progression are recapitulated. More detailed characterization of the inter-batch and intra-batch organoid heterogeneity on parameters such as the proportion of each cell type within the organoids and functional maturity of each cell type would also be required. 

Employing a similar stepwise PSC differentiation approach, Ramli et al. created a hepatic organoid consisting of mainly parenchymal cells for modeling drug-induced liver injury and MAFLD [47]. The parenchymal cells in the organoid interact during the differentiation process to form a bile canaliculi network in the hepatocyte core and connect to the cyst-like structures formed by the cholangiocytes in the periphery [152]. This structural feature in the liver organoid enabled the authors to model tissue architecture changes during MAFLD progression. Through multiphoton imaging of MAFLD patient liver tissues, Segovia-Miranda et al. reported that the bile canaliculi network in early-stage and late-stage patients is progressively diminished [153]. The authors were able to model this phenotypic change with the FFA treatment of the organoids. In addition, FFA treatment increases the proportion of CK7+ cholangiocytes in the organoids, which resembles the ductular proliferation observed during NASH progression (Figure 3B). This was further supported by the increased number of Ki67+ cholangiocytes detected in the FFA-treated organoids. The study highlights the potential of liver organoid models in capturing tissue-level pathophysiology during MAFLD progression, which was largely dependent on animal model studies. A primary limitation of this model is the lack of NPCs which hinders the further modeling of inflammatory and fibrogenesis development during NASH progression. 

Multi-cellular liver models can also be assembled by co-culturing different cell types (Table 2). PSC-derived liver organoids are composed mainly of cells of fetal nature which may not recapitulate the full function of the mature adult cells. The co-culturing approach provided the flexibility to use only functionally mature cell types to overcome this issue. In addition, the co-culture approach enables precise control of the proportion of different cell types within the 3D cell model. This reduces the heterogeneity of organoids generated across batches, favoring drug testing and screening applications. These advantages were well demonstrated by the in vitro microtissue model reported by Ströbel et al., where the team co-cultured primary human hepatocytes, Kupffer cells, liver endothelial cells, and HSCs [154]. Human liver microtissues (hLiMTs) were cultured in suspension devoid of scaffolds, and MAFLD modeling was achieved with a 10-day treatment regime using NASH-inducing media composed of elevated glucose, fructose, and FFA. The authors further induced inflammatory response using a short pulsing treatment with LPS. The hLiMTs progressively exhibited hallmarks of early and late MAFLD, including steatosis, inflammation, ballooning, and fibrosis. The homogeneity of the NASH hLiMTs enabled the authors to utilize the system to demonstrate the therapeutic effect of NASH drugs targeting different MAFLD phenotypes: the anti-steatotic and anti-fibrotic effects of Firsocostat, the anti-inflammatory and anti-fibrotic effects of Selonsertib, and the anti-fibrotic effect of ALK5i. Despite these advantages, the direct co-culture of the different liver cell types in suspension or matrices did not result in the formation of structures resembling the liver tissue. In most reported co-culture approaches [47,50,126,154,155,156,157] (Table 2), cells were homogenously mixed and randomly distributed across the 3D liver model formed. In contrast, the PSC differentiation process to generate organoids recapitulates morphogenesis events during embryonic development. During this process, cells respond and form interactions and structures according to changes in environmental cues. 

**Table 2:** Human multi-cellular 3D MAFLD models.

Key Approach	Cell Culture Method	Major Features
**Self-organizing liver organoid models**	**Step-wise differentiation from pluripotent stem cells (PSC)**	PSC-derived organoids that are composed of multiple cell types, including hepatocyte-like cells, Stellate-like cells, and Kupffer-like cells [50]. Organoids exhibit steatosis, inflammation, and fibrosis response upon free fatty acid (FFA) treatment [50]. Increased organoid stiffness recapitulated in vivo liver fibrogenesis event and was employed for drug response study [50]. Inter and Intra batch variability observed. Further characterization of biochemical changes in cells during FFA treatment is required [50].
		PSC-derived liver epithelial organoids that are expandable and can differentiate into hepatocytes [126]. Epithelial organoid-derived hepatocytes readily take up FFA and accumulate lipid droplets, enabling the testing of various drugs for reducing steatosis [126].
		PSC-derived organoids that are primarily composed of hepatocytes at the core and cyst-forming cholangiocytes in the peripheral [47]. Structural features in liver organoid enabled modeling of tissue architecture changes in the liver during MAFLD progression, including bile canaliculi network disruption and ductular reaction [47]. Organoids lack non-parenchymal cell types, which limits modeling of inflammation and fibrogenesis [47].
	**Co-culture of parenchymal and non- parenchymal liver cell types**	Co-culture of different hepatic and non-hepatic cells to form 3D spheroid cultures in suspension [154,155,156] or matrices [157]. The majority of these co-cultured spheroids do not recapitulate liver tissue structure [154,155,156,157]. The inclusion of fibroblast and stellate cell lines enable the modeling of fibrogenesis event, and the inclusion of Kupffer cells allow the modeling of inflammatory events [154,155,156,157]. The direct co-culture of mature functional cell types enabled better control of cell type proportions to achieve higher homogeneity and reproducibility of organoids for quantitative applications, especially in drug testing [154,155,157]. This approach enables genetic manipulation of selected cell populations before co-culture to enable cell-type specific targeting.
**Bio-engineered liver models**	**Microfluidics culture**	Culture composed of largely immortalized hepatic cells [79,158] or primary hepatocytes [159]. Microfluidics enabled cell culture with circulation to mimic vascular flow [79,158,159]. The system facilitates the continuous exchange of molecules, including nutrients and metabolites, which mimics physiological conditions during MAFLD development. The introduction of vascular flow enhances cellular function compared to static cultures [79,158,159]. The media flow also improves cell viability in the core of hepatic spheroid [79].
		The control of system parameters achievable with the microfluidics platform enables the seeding of multiple cell types together [51,160,161,162], or in separate chambers [162] within a chip. The use of microfabrication techniques enables precision placement of cells [162]. Separation of cells in chambers with porous walls mimics the vascular system. It also enables the layering of cells to achieve a similar spatial arrangement of cells observed in the liver tissue [163]. Incorporation of PSC differentiation approach in microfluidics platform enables generation of organoids-on-a-chip [51]. Manipulation of chip configuration enables recapitulation of liver lobule distribution of cells to mimic in vivo tissue organization [160]. Limited throughput and the requirement of specialized equipment and techniques limit the wide adoption of microfluidics platforms.
	**Precision Cut Tissue Slice (PCTS)**	PCTS enables the direct use of patient tissue from biopsy for drug response studies [164]. PCTS maintains intact cellular interactions and organizations observed in MAFLD liver tissues, which may be favorable for drug response study [164]. The short-term culture may also enable the capture of host-pathogen interactions that may influence drug treatment response [164]. Limited application due to short culture period (only up to 5 days) and availability of human tissue [164].
	**Organ scaffolds**	Co-culture of iPSC-derived HLC, HUVEC, mesenchymal stromal cells, fibroblast, and blood-derived macrophages in decellularized liver tissue [165]. One of the largest centimeter-size liver organoids cultured. Authors employ a peristaltic pump system to deliver nutrients to the core of the tissue. This enhanced the viability of cells and penetration of MAFLD phenotype throughout the tissue [165]. The tissue-like culture exhibit MAFLD hallmarks which enabled steatosis, inflammation, and ballooning scoring comparable to patient tissue [165].

## 4. Bioengineered Liver Models

One of the main physiological aspects lacking in the cellular models discussed thus far is the vascular systems in the liver tissue. The vascular system is essential for modeling multiple MAFLD features (Figure 2A and Figure 3A), including immune cell migration in the liver sinusoid during inflammation and fibrogenesis. Bioengineering techniques are often employed in recreating such a vascular system which is also crucial for supplementing nutrients to cells in the core of large 3D cellular models [166,167,168,169]. In addition, bioengineering techniques are also often engaged to achieve the precision placement of cells in a multi-cellular model. One of the main approaches includes using microfluidics with compartments and molecular scaffolds that allow controlled seeding and layering of cells to mimic the liver sinusoid. Furthermore, more sophisticated bioengineering approaches such as bioprinting are employed to achieve precision placement of cells on a chip. Moreover, organ scaffolds are often utilized to create large centimeter-sized cellular models. 

### 4.1. MAFLD Models with Microfluidics Platform

The microfluidics platform has been extensively employed in the organ-on-a-chip system, which aims to recreate human tissue physiological models [170,171]. The complexity ranges from single-cell-type culture to multiple-cell-type co-culture in one or more chambers joined by continuously perfused microchannels. The flow system aims to recapitulate tissue- and organ-level relevant fluid shear stress and achieve constant exchange of fluids and biomolecules. The microfluidic system can recapitulate mechanical stretch and compression observed in the tissues by employing different chip designs and materials, including cell culture matrices. A distinct advantage of this bioengineering approach to generating human liver models is the control of system parameters achievable compared to other described strategies. This includes control of cell–cell interaction and cell–matrix interaction achieved by chamber partitioning and selective matrix deployment, control of speed and direction of fluid flow, and monitoring cell status with microsensors incorporated in the chips. Liver-on-a-chip is widely utilized for drug efficacy and toxicology studies [79,172,173,174]. 

To recreate the liver sinusoid, Feaver and colleagues co-cultured primary hepatocytes, Kupffer cells, and HSCs in a transwell system that incorporated a cone-and-plate viscometer and an independent flow system [163]. The setup recapitulates the vascular flow dynamics and allows a continuous media exchange. The hepatocytes were sandwiched in collagen and separated from the Kupffer cells and HSCs by a porous membrane, enabling direct cell–cell interactions and indirect interactions through secreted factors. More importantly, separating the NPC from the hepatocytes in the setup enabled the measurement of cell-type-specific responses. The authors were able to specifically look at the transcriptome changes in hepatocytes and correlate the changes with the secretome profile, which also contains signals from the NPC. Using the system, the authors validated that the hepatocytes’ response to the lipotoxic milieu was independent of the NPC. Furthermore, the authors validated potential biomarkers in effluent from the cells at multiple time points during the ten days of NASH-inducing conditions. While the use of collagen sandwich culture hindered the measure of collagen secretion for quantifying fibrogenesis response, the authors could detect pro-fibrogenesis molecules such as TGF-β and OSTEOPONTIN in the effluent. Correspondingly, HSCs under NASH treatment conditions exhibit significant morphological change and enriched α-SMA expression. The study further demonstrated the utility of this MAFLD model for assaying obeticholic acid (OCA) treatment responses.

Multiple groups have similarly utilized the microfluidics platform to create MAFLD models harboring hepatic cells [79,158,159] or co-cultured with NPCs [51,160,161,162,174]. Taking a step further, Wang et al. demonstrated the feasibility of growing PSC-derived liver organoids in the microfluidic system, highlighting the potential of integrating different approaches for creating MAFLD liver models [51]. In another study, Davidson et al. showed that microfabrication techniques could be employed to achieve precision placement of hepatocytes and NPCs on a chip [162]. Using the micropatterned co-cultures (MPCCs) approach pioneered by Bhatia’s group [175], the authors achieved precision placement of the HSCs around islands of hepatocytes. They demonstrated the utility of the scalable platform for modeling NASH and drug testing. While the microfluidic approach resolved several issues in modeling liver disease in vitro, the relatively low throughput, high cost, and limited materials for downstream molecular studies compared to other cell culture methods restricted the applications of the system. It will also be of challenge to scale the system for high-throughput drug screening study, given that the enclosed flow system limits the number of drugs that can be separately supplemented to each MAFLD model on the chip.

### 4.2. MAFLD Tissue-like Culture with Liver Tissue Slices and Organ Scaffolds

An intriguing cell culture approach for modeling human diseases is the direct use of microtissue slices from patient biopsies [176]. Precision-cut tissue slices (PCTSs) of up to 0.5 cm in diameter and 250 µm thick (10–15 cell layers) are trimmed from the biopsies and maintained in a culture media for up to 5 days. Well-processed PCTSs maintained the cellular interaction, spatial organization, and structural features of liver tissue and harbored the disease’s pathophysiological aspects. In addition, the tissues may contain host–pathogen interactions that could modulate treatment responses in each patient. Hence, PCTSs closely resemble in vivo human tissues and provide a physiological and patient-relevant model for drug studies. Harnessing this unique advantage of PCTSs, Ijssennagger and colleagues generated the first direct gene expression profile induced in the liver tissue upon OCA treatment [164]. The authors generated PCTSs from liver biopsies of three histologically proven NASH patients and treated the PCTSs with OCA for 24 h before harvesting them for global gene expression profiling. Corresponding to the role of OCA as a farnesoid X receptor (FXR) agonist, the top candidates in the differentially expressed gene list include FXR target genes, including *FGF19*, *NR0B2*, *ABCB4*, and *ABCB11*. In parallel, the authors profiled liver tissues from wild-type and FXR-/- mouse models, and cross-examination of genes identified in both the PCTSs and mouse liver identified a novel list of OCA downstream genes. A major disadvantage of the system is that the PCTS can only be cultured for an average of 5 days [176]. Moreover, the system is only available when patients undergo surgery for liver biopsy. Needle biopsy procedures pose a significant health risk for MAFLD patients [177], which limits the number of patients willing to undergo the process for complete MAFLD diagnosis. 

An alternative approach to harness human and mammalian liver organs and tissue to generate an organ-like culture in vitro is the use of decellularized organ scaffolds [165,178,179]. The organs and tissues of human or animal origins are first decellularized enzymatically, and a scaffold containing largely ECM remains. Primary or immortalized human cells are introduced into the scaffolds, and the existing ECM supports cell adhesion and repopulation. This approach harnesses the endogenous architecture of the tissue to recreate organ-like 3D cultures with cellular distribution and interactions mimicking the in vivo tissues. Utilizing this approach, de l’Hortet et al. generated one of the largest centimeter-size human NASH models [165]. The team repopulated liver scaffolds from decellularized rat liver with genetically modified PSC-derived HLCs, mesenchymal cells, fibroblasts, and Kupffer cells. A perfusion pump was employed to systematically introduce the cells to achieve an even distribution throughout the scaffold and to deliver nutrients and drugs throughout the organ-like structure. The setup enables the penetration of the NASH phenotype throughout the entire organ-like culture compared to static organoid culture (prepared using co-culture of similar cells), where the phenotype is only limited to the edges. Remarkably, the tissue-like organ enabled NAS scoring for the MAFLD hallmarks, including steatosis, inflammation, and ballooning, comparable to NASH patient tissues. On the other hand, it remains to be seen if this organ-like culture system could incorporate HSCs, which is crucial for the deposition of the ECM essential for fibrogenesis modeling. It is also unclear how drug response in such a centimeter-size organ would resemble in vivo therapeutic response more closely. 

## 5. Current Challenges and Future of MAFLD Modeling

### 5.1. Modeling Immune Landscape of MAFLD Progression

The liver is a highly vascularized organ with abundant migrating cells of both the innate and adaptive immune systems. The roles of each immune cell type in NASH development and progression were well illustrated in the review by Huby and Gautier [180]. In contrast, immune cells are lacking in most studies highlighted, and only macrophages or Kupffer cells were employed in some studies. The chronic inflammatory response is the underlying driver of MAFLD progression and is mainly induced by the injured hepatocytes and circulating inflammatory cues from the gut and adipose tissue [181]. Innate immune cells such as the resident Kupffer cells, neutrophils, and circulating monocytes play a crucial role in mediating this response. Drugs antagonizing CCL2 and CCL5 receptors to reduce macrophage recruitment to the liver have shown efficacy in reversing NASH phenotype in preclinical models and phase II clinical trials [52]. On the other hand, a significant fraction of obese patients with steatosis do not progress to the NASH stage, and the time taken to develop NASH is lengthy and highly variable. This observation suggests that the innate immune cells in the liver of these patients are likely non-inflammatory, or their inflammatory responses are nullified. Single-cell profiling of tissues from obese patients with steatosis supports the former [182]. Single-cell profiles of healthy and diseased liver tissues have further highlighted the existence of up to seven macrophage populations, including the previously described pro-inflammatory or pro-restorative proliferative (anti-inflammatory) cell states [183,184]. Understanding how these different cellular states are modulated in the steatotic liver and their exact role in mediating inflammatory response and fibrogenesis may be crucial to unravel the phenotypic diversity in NASH progression among MAFLD patients. Human in vitro MAFLD organoid models that could support these diverse macrophage cells would greatly facilitate such mechanistic studies. 

While most MAFLD models have focused on supplementing Kupffer cells to model inflammatory response, there is growing evidence that different immune cell types are implicated in MAFLD development and progression [181,185]. Lymphocyte infiltration into the liver lobules has been a histological feature of NASH [2], and aggregates of T and B cells can be detected in most NASH patient biopsies [186]. Increased lymphocyte infiltration in the portal region is also closely associated with ductular reaction in NASH patients [54]. Selective depletion of CD4+ memory T cells or CD8+ cytotoxic T cells reportedly inhibits NASH progression in mouse models [187]. So far, the role of these immune cells in NASH has been limited to observational studies in vivo, and how the T cells interact with other cell types is limited due to a lack of in vitro models. It remains unclear how the different selective populations of T cells are enriched in the NASH microenvironment and how their activity modulates inflammatory responses and fibrogenesis. While most studies have focused on the roles of immune cells in driving inflammation and fibrogenesis events during MAFLD progression, there is growing interest in how the resolution of the NASH features is impeded [188]. Besides the macrophages, neutrophils are emerging as an important immune cell type that mediates both NASH progression and resolution [189]. Kim et al. demonstrated that the selective depletion of neutrophils at the restorative phase (high-fat-diet removal) inhibits the resolution of inflammation and fibrogenesis in high-fat-diet mouse models [189]. The population of pro-restorative macrophages was significantly reduced, and HSCs remained highly activated when neutrophils were depleted in MAFLD mice under restorative conditions. Unraveling how the neutrophils mediated such responses would be fundamental to designing therapeutic strategies that improve the resolution of NASH features. In summary, the emerging roles of different immune cell types in MAFLD signal the requirement for increasingly complex systems that support more diverse immune-cell-type co-culture with liver cells. There may also be a need to spatially organize the cells to mimic key immunogenic events such as the immune infiltration observed during different stages of MAFLD progression. 

### 5.2. Recapitulating MAFLD Stage Transition and Structural Features of Late MAFLD Development

A significant limitation of MAFLD mouse model studies is the lack of tools for monitoring critical disease stage transition events. How the inflammatory responses and fibrogenesis in the liver occur to remodel the liver niche gradually remains vague. Most in vivo studies only provide a snapshot of the disease status, and gaps of knowledge on disease progression across stages remain. Human in vitro models can provide a continuum observation of how MAFLD progresses or transits between stages. However, few or none of the studies discussed have been able to describe stepwise treatment conditions or demonstrate a time-lapse of treatment that could demarcate MAFLD stage transition. In most studies, steatosis, inflammatory response, and fibrogenesis were captured in parallel at specific treatment timepoints [50,163,165]. None of the co-culture and organoid studies have developed a dynamic culture system where steatosis without activation of the immune cells and HSCs can be observed under fatty liver culture conditions. The lack of control over disease stage progression limits the full potential of human models for MAFLD disease studies. Achieving this breakthrough would likely require using microfluidic platforms for precise control of the cellular spatial organization, interactions, and exposure to environmental change. The successful modeling of MAFLD transition events would be of great value to unraveling the molecular interplays resulting in the lengthy period observed for NASH progression. The model will also be critical for identifying biomarkers specific to NASH development and reversal. Liver biopsy remains the most relied upon and recognized diagnosis method for NASH evaluation. This requirement slows patient recruitment and poses challenges for monitoring NASH reversal during clinical trials [190]. Human models of MAFLD progression would serve as an excellent evaluation model for many of the biomarkers identified in tissue profiling studies from multiple patient cohorts [16,19,49]. 

**Figure 4:**
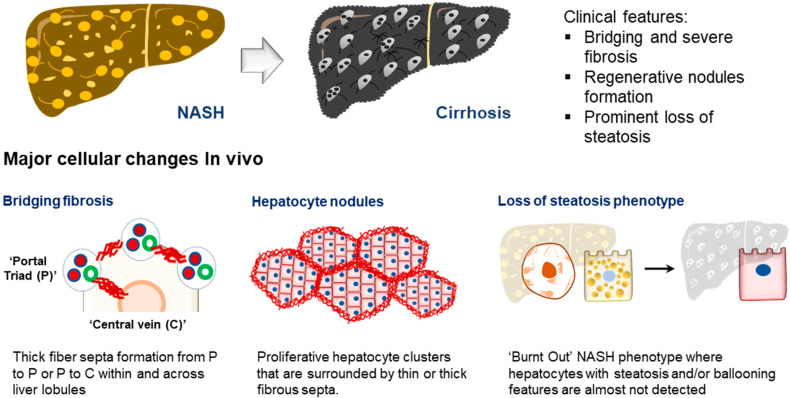
**Modeling MAFLD-induced cirrhosis**. (Top) Tissue features observed in liver biopsies from patients with cirrhosis. (Bottom) The major molecular and cellular changes observed in MAFLD patients with liver cirrhosis.

While we observe significant progress in modeling tissue architecture changes in NASH progression in some of the studies highlighted, it is evident that key structural changes during the late stage of NASH progression, especially at cirrhosis stages, are absent (Figure 4). In the same context, diet and genetic mouse models of MAFLD have been extensively reported [191], and NASH progression to cirrhosis was only reproducibility observed in non-physiologically relevant choline-deficient diets [192]. Advanced fibrosis with a score greater than F3 has only been observed in diet-induced obesity mice treated up to 40–50 weeks. Fibrogenesis observed in most abovementioned human models likely recapitulates the perisinusoidal fibrogenesis occurring in the early MAFLD stage. Human MAFLD fibrogenesis models with bridging fibrosis in late disease development have yet to be reported. Such models would likely require a human cell culture system that can sustain for an extended period of up to months to enable the buildup of thick fibers septa observed in bridging fibrosis [193,194,195]. Current clinical trials require patients to have a NAS score of 4 or more and greater than stage 1 fibrosis where bridging fibrosis is observed [196]. Regression of fibrosis remains a key performance indicator for clinical trials, and in many cases, the resolution of bridging fibrosis remains a crucial challenge. During cirrhosis development, regenerative nodules of hepatocytes surrounded by fibrous tissue are also observed [193,194,195,197]. Such nodules can be either benign nodular hyperplasia or dysplastic nodules with tumorigenic potential, underlining the transition to HCC development. Intriguingly, in the biopsy of MAFLD patients that eventually develops cirrhosis, there is a near complete loss of steatosis and ballooning phenotype [198]. This contradictory observation poses questions about whether similar lipotoxicity and inflammatory events drive the final stages of NASH progression and when alternative mechanisms are initiated. The mechanism driving these mentioned phenotypes remains largely unknown due to the lack of human models. 

### 5.3. Modeling Organ Crosstalk in MAFLD

Organ crosstalk plays a significant role in MAFLD development and comorbidities. For instance, the enrichment of specific microbial species in MAFLD patient guts [28,30] supports liver–gut axis crosstalk, where gut microbiome metabolites regulate MAFLD development and progression. Such influence is further observed in patients with leaky gut syndrome [199]. These patients have deteriorating gut walls where the gap junctions between the epithelial cells are damaged and lose their partitioning function, facilitating the transport of PAMPs and metabolites across the hepatic portal vein into the liver. Besides the gut, multiple tissues have been reported to influence MAFLD development or strongly correlate with MAFLD severity, including adipose tissues, pancreas, muscle, brain, and thyroid [200,201]. On the other hand, MAFLD has been reported to be an independent factor of diseases in other organs, especially heart and kidney diseases [1,202]. Considering these influences by other organs during NASH progression and comorbidities, future MAFLD models will likely need to incorporate these cells or tissues for investigating multiple organ crosstalk.

## 6. Concluding Remarks

A plethora of human MAFLD models, from monolayer single-cell-type cultures to advanced 3D tissue-like cells, have been created. MAFLD features recapitulated range from lipid buildup and metabolic changes in the hepatocytes (Figure 2B) to inflammatory response and fibrogenesis events, which require crosstalk of both liver parenchymal and NPCs (Figure 3B). Each model discussed is advantageous for different applications. Human MAFLD models are created for mechanistic studies and high-throughput drug screening applications that require a large number of materials and for studies unraveling liver structural changes, which need multi-cellular interactions and tissue-like organization of the cell types. Multiple challenges have been highlighted in these studies, and breakthroughs would likely require advancement in cell culture methods and media to preserve multiple cell type functions. In parallel, bioengineering approaches would be essential to achieve the co-culture of liver parenchymal and NPCs in physiologically relevant spatial organization. In most of the studies presented, the application of human MAFLD in vitro models remains limited to the recapitulation and validation of observation in mice and patients and mechanistic studies. Improvements to future models that better reflect MAFLD liver tissue would be essential to expand their applications, including screening for novel MAFLD therapeutics.

## Figures and Tables

**Table 1:** Human primary and immortalized hepatic cells used for modelling MAFLD.

Cell Type	Cell Lines	Major Features
**Immortalized Cell lines**	**HepG2**	One of the earliest in vitro cell models for recapitulating MAFLD development in hepatocytes [43]. Treatment with unsaturated free fatty acids (FFA) such as oleic acid induces hallmarks of early MAFLD development, including elevation of intracellular triglyceride levels, lipid micro-vesicle and macro-vesicle formation, increased lipid peroxidation, and reduced cell viability [43,44,69,72,73,74]. Treatment with saturated FFAs such as palmitic acid further enhances lipid accumulation, changes in oxidative phosphorylation, and increased cell apoptosis and ER stress responses [44,73,74]. The ease of manipulating immortalized cell lines facilitates loss and gain of function studies to unravel mechanisms of drug response and MAFLD development [75]. Cancer origin of cells and molecular changes introduced by the immortalization process are concerns for physiological relevance of such cell line models.
	**Huh7**	Huh7 accumulates a much higher level of triacylglycerols (TAGs) compared to HepG2 when exposed to bovine or human Serum, highlighting the diverse fatty acid metabolic activity across different cell lines [69,76].
	**WRL68**	Cells treated with FFAs develop similar steatosis phenotypes in comparison with HepG2 [77].
	**HepaRG**	Cells exhibit greater sensitivity to drug-induced steatosis compared to HepG2 [78]. Cells treated with FFAs develop similar steatosis phenotypes compared to HepG2 [79].
**Tissue-derived primary cells**	**Primary Hepatocyte (PHH)**	PHH derived from human liver tissue remains the most physiologically relevant hepatocyte cell model. However, usage in the modeling of human liver diseases has been limited due to donor availability. Immortalized PHH could potentially provide a renewable source of human hepatocytes for MAFLD studies [80]. MAFLD phenotypes could be induced with treatment using FFA (oleic acid, palmitic acid, and stearic acid) and fructose [41,44,45,70,80,81,82]. Conditioned media from hepatocytes treated with FFA with and without fructose can induce fibrogenic responses in hepatic stellate cells [45,70]. 3D spheroid culture of PHH improves hepatocyte function and maintenance compared to 2D culture and is favorable for chronic MAFLD modeling [41,82]. Hepatocytes from donors harboring previously reported TM6SF2 E167K genetic variant exhibit increased lipid accumulation under FFA treatment compared to other donors [82]. This study highlights the value of patient-derived hepatocytes in evaluating genetic risk variants identified in genome-wide association studies.
	***Bipotent ductal stem cells***	Derivation of bipotent ductal stem cells from NASH patient tissue biopsies using organoid culture platform [46]. Upregulation of pro-inflammatory pathway genes, cytochrome p450-related pathways genes, and genes associated with fibrogenesis and tumorigenesis in specific NASH patient-derived ductal organoids [46]. Differentiated NASH patient-derived organoids exhibit enhanced NASH phenotypes compared to healthy controls [46]. Patient-specific idiopathic response, similar to MAFLD studies using PHH models, was observed [46].
**Pluripotent stem cells (PSC)-derived primary cells**	**Hepatocyte-like cells (HLC)**	Induced PSC (iPSC) technology enables the establishment of patient-specific MAFLD models for precision therapeutic studies [19,49]. HLCs generated from NASH patient-derived iPSC express disease signatures observed in patient tissues [49]. The ease of genetic manipulations of iPSC and capturing of patient-specific genotypes facilitate the generation of human genetic MAFLD models [19,49]. Self-renewing PSCs enable the generation of large numbers of primary HLC cells for downstream molecular profiling [19,48,49,83,84], and drug screening experiments [83]. Fetal nature of PSC-derived cells remains a concern for physiological relevance.

## Data Availability

Not applicable.

## Abbreviations

**List of Abbreviations Used**			
**Abbreviation**	**Full Name**	**Gene Name**	**Full Name**
2D	2-dimensional	*ABCB11*	ATP Binding Cassette subfamily B member 11
3D	3-dimensional	*ABCB4*	ATP Binding Cassette subfamily B member 4
AAA	Aromatic Amino Acid	*ALB*	Albumin
BCAA	Branched-Chain Amino Acid	*APOC3*	Apolipoprotein C3
BMP-8B	Bone Morphogenetic Protein-8B	*CK7*	Keratin 7
CCL	C-C motif Chemokine Ligand	*CK8*	Keratin 8
CDK	Cyclin-dependent Kinase	*CLEC4F*	C-type lectin domain family 4 member F
CKD	Chronic Kidney Disease	*CYP3A4*	Cytochrome P450 family 3 subfamily A member 4
CTRP9	C1q/TNF-Related Protein 9	*FGF19*	Fibroblast Growth Factor 19
CVD	Cardiovascular Disease	*GCKR*	Glucokinase Regulator
CXCL	Chemokine (C-X-C motif) Ligand	*HNF4A*	Hepatocyte Nuclear Factor 4 alpha
DAMP	Death-associated Molecular Pattern	*HSD17B13*	Hydroxysteroid 17-beta Dehydrogenase 13
DNL	De Novo Lipogenesis	*MBOAT7*	Membrane-Bound O-acyltransferase Domain-Containing 7
ECM	Extra-cellular Matrix	*NR0B2*	Nuclear Receptor subfamily 0 group B member 2
ER	Endoplasmic Reticulum	*PNPLA3*	Patatin-like Phospholipase Domain-Containing 3
FFA	Free Fatty Acid	*TM6SF2*	Transmembrane 6 Superfamily Member 2
FXR	Farnesoid X Receptor		
GLP1R	Glucagon-Like Peptide 1 Receptor		
GWAS	Genome-wide Association Studies		
HCC	Hepatocellular Carcinoma		
HLC	Hepatocyte-like Cell		
hLiMT	Human Liver Microtissues		
HSC	Hepatic Stellate Cell		
IL	Interleukin		
iPSC	Induced Pluripotent Stem Cell		
IRS2	Insulin Receptor Substrate-2		
LSEC	Liver Sinusoidal Endothelial Cell		
MAFLD	Metabolic (Dysfunction) Associated Fatty Liver Disease		
MMP	Matrix Metalloproteinase		
NAFLD	Non-alcoholic Fatty Liver Disease		
NASH	Non-alcoholic Steatohepatitis		
NEFA	Non-esterified Fatty Acid		
NK	Natural Killer		
NPC	Non-parenchymal Cell		
OA	Oleic Acid		
OCA	Obeticholic Acid		
P3NP	N-terminal pro-peptide of collagen type III		
PA	Palmitic Acid		
PAMP	Pathogen-associated Molecular Pattern		
PCTS	Precision-Cut Tissue Slice		
PDGF	Platelet-Derived Growth Factor		
PHH	Primary Human Hepatocyte		
PI3K	Phosphatidylinositol-3-OH Kinase		
PKC	Protein Kinase C		
PSC	Pluripotent Stem Cell		
ROS	Reactive Oxygen Species		
SA	Stearic Acid		
SGLT2	Sodium–Glucose Cotransporter-2		
SNP	Single-Nucleotide Polymorphism		
T2DM	Type II Diabetes Mellitus		
TH17	T helper 17 (TH17) cells		
TGF	Transforming Growth Factor		
TNF	Tumor Necrosis Factor		
Treg	Regulatory T cells		
VLDL	Very-low-density lipoprotein.		
α-SMA	Alpha-Smooth Muscle Actin		
